# How much schizophrenia do famines cause?

**DOI:** 10.1038/s41537-023-00416-2

**Published:** 2023-12-19

**Authors:** Cormac Ó Gráda, Chihua Li, L. H. Lumey

**Affiliations:** 1 University College Dublin, Dublin, Ireland; 2 Institute for Social Research, University of Michigan, Ann Arbor, MI USA; 3 Department of Epidemiology, Mailman School of Public Health, Columbia University, New York, NY USA

**Keywords:** Diseases, Schizophrenia

## Abstract

Since the 1970s, famines have been widely invoked as natural experiments in research into the long-term impact of foetal exposure to nutritional shocks. That research has produced compelling evidence for a robust link between foetal exposure and the odds of developing schizophrenia. However, the implications of that research for the human cost of famines in the longer run have not been investigated. We address the connection between foetal origins and schizophrenia with that question in mind. The impact turns out to be very modest—much less than one per cent of the associated famine death tolls—across a selection of case studies.

## Introduction

Several academic studies have linked the current type 2 diabetes (T2D) epidemic in China to the Great Leap Forward famine of the 1950s^1–5^. In Ireland, a rise in the prevalence of mental illness—and schizophrenia, in particular—in the second half of the nineteenth century has similarly been blamed on the Great Irish Famine^6,7^. Much in the same vein, historian and geographer Jared Diamond^8^ has claimed that we ignore the lessons of the Dutch Hunger Winter of 1944–45 “only at our children’s, and our grandchildren’s, expense”. Further afield, drought and famine in the Horn of Africa in the recent past have been tied to “a potential scenario of new ‘hot spots’ for type 2 diabetes”^3^. Beyond academia, such claims have a broad appeal. They have prompted online advice to women not to try to lose weight during pregnancy, and warnings such as that famines about famines accelerating brain ageing, boosting cancer risks, and making future generations obese.

All these claims are prompted by research that uses famines as natural experiments, in order to test versions of the hypothesis that “chronic, degenerative conditions of adult health … may be triggered by circumstance decades earlier, *in utero* nutrition in particular”^9^. Unintentionally, they have created an impression that the post-famine footprint of foetal exposure was substantial. Is that really the case? This paper focuses on the odds of developing schizophrenia in adulthood following foetal exposure as a means of answering this question. On the basis of several well-known case studies, it compares the size of what might be called ‘the foetal origins effect’ to the short-run cost of famine in terms of lives lost.

Since the pioneering work of Stein et al.^10^ on the Dutch Hunger Winter of 1944–45, famines have often been invoked as natural experiments to test versions of the hypothesis that foetal exposure exacts a cost in terms of adverse health outcomes in later life. Since the 1990s, especially, the role of famines as human laboratories to study biomedical mechanisms has been widely recognised. Furthermore, as interest has grown in the broader long-term consequences of foetal exposure for economic and social wellbeing, the influence of this research has expanded beyond epidemiology into the social sciences. For economic and demographic historians, this research is a reminder that famines had an afterlife; they inflicted physical and mental damage in adulthood on the health of some of those born during them or in their immediate wake. In this study, we will use the number of schizophrenia cases attributable to *in-utero* famine exposure as a measure of the long-term cost of famine.

## Schizophrenia, foetal origins, and famine

Schizophrenia is a severe and chronic mental illness. Notwithstanding significant advances in treatment, it remains a major public health issue. It is the cause of significant psychiatric morbidity, and exacts a big penalty in terms of reduced life expectancy, with a recent meta-analysis putting the average number of years of life lost at 15^11–16^. While schizophrenia has an important genetic component, its triggers may include disparate factors such as drug use, malnutrition, stress, and early-in-life trauma. Yet although research on the link between exposure to large-scale natural disasters and wars, on the one hand, and psychiatric illness, on the other, is abundant, that literature has not focused so far on the onset of schizophrenia specifically^17,18^.

We focus on schizophrenia here because one of the most robust findings of the foetal origins literature is that exposure in early gestation roughly doubles the odds of schizophrenia in adulthood among both males and females. This finding first emerged from research using the Dutch Hunger Winter as a case study^19–22^. It was soon replicated in a 2005 study using data from the severely affected Chinese province of Anhui during the Great Leap Forward famine of 1959–61^23^. Subsequent research on China corroborates^24–27^. The outcomes are given in Table 1, where CI refers to a 95% confidence interval. In what follows we assume that foetal exposure during famines doubled the odds of developing schizophrenia.

**Table 1:** Foetal origins and the odds of Schizophrenia.

Study	Country and date	Odds ratio
Susser et al.^20^	Netherlands 1944–45	2.0 (95% CI: 1.2–3.4)
St. Clair et al.^23^	China 1959–61	2.30 (95% CI: 1.99–2.65) (births in 1960) 1.93 (95% CI: 1.68–2.23) (births in 1961)
Xu et al.^24^	China 1959–61	1.68 (95% CI: 1.48–1.92) (births in 1960) 25 (95% CI: 2.00–2.52) (births in 1961)
He et al.^25^	China 1959–61	1.82 (95% CI: 1.11–2.98)

The odds ratio is the ratio of an event occurring in a treatment group to the odds of an event occurring in a control group.

**Fig. 1:**
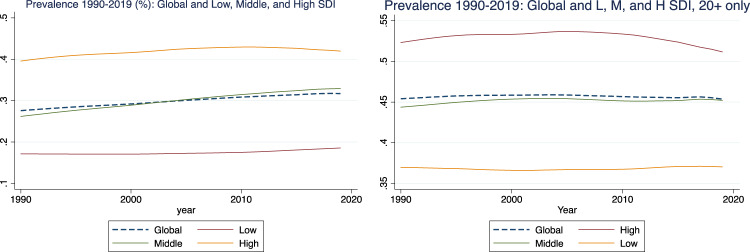
Prevalence of schizophrenia 1990–2019. Source: Our World in Data (https://ourworldindata.org/grapher/schizophrenia-prevalence).

Knowing the prevalence of schizophrenia in the population at large (*p*) is also essential for assessing the impact of foetal exposure during famines. According to the World Health Organisation (WHO) schizophrenia today affects “approximately 24 million people or 1 in 300 people (0.32%) worldwide”^15^, the prevalence rate being 0.45% among adults. The WHO data imply that prevalence is related to country income; in 1990, in high-income countries it was 0.38%, whereas in low-income countries it was only 0.16%. In 2019 the percentages were 0.41 and 0.17, respectively. To what extent the differences reflect the quality of recording, diagnosis, and institutionalisation is unclear; other evidence seems to suggest that poverty is a strong predictor of schizophrenia^28^. However, the data imply that the prevalence of schizophrenia has been relatively stable in recent decades (Fig. 1). This contrasts with the prevalence of some other disease outcomes, such as obesity and Type 2 diabetes, which have become much more common. Age-standardised prevalence data are available globally only since 1990 (most conveniently at *Our World in Data*), later than the years covered in this study. For that reason, and also in order to err on the side of generosity (for a reason explained below), we work with an assumed *p* of 0.5% (Fig. 1).

Calculating the impact of famine on the number of additional schizophrenia cases in adulthood requires estimates of the increase in the odds of developing the disease (*d*), the prevalence of the disease in normal times (*p*), and the number of births at risk due to foetal exposure (*B*). Given the availability of data on *d*, *p*, and *B*, producing an initial upper-bound estimate of the cost of exposure during a famine as *B*.*d*.*p* is straightforward. The estimate is upper-bound because it makes no allowances for deaths in childhood and adolescence to the cohorts at risk^29^. We return to this issue below. In order to contextualise and provide an informal metric for the resultant welfare loss, we compare it to the total number of lives lost in the famine in question.

## Method: five famine settings

This section briefly describes five well-known famines, focusing on estimates of excess mortality and live births prenatally exposed to famine. These famines, which differed substantially in severity, duration, and magnitude, are the Dutch Hunger Winter of 1944–45; the Leningrad Blockade Famine of 1941–43; the Great Ukraine Famine of 1932–33; the Great Irish Famine of 1845–1850; and the Chinese Great Leap Forward Famine of 1959–61. The impact of famine on deaths and births in each of these cases is discussed in reverse chronological order.

### China 1951–61

The Chinese Great Leap Forward famine of 1959–61 lasted for around three years. While estimates of excess mortality are still contested, a figure of about 30 million is the most often mentioned^30^. During the famine, the birth rate declined from 35 per 1000 to 20 per 1000; population censuses conducted during the 1980s imply that births exposed to famine in utero were 35–40 million. We accordingly assume *B* = 40 million.

### The Netherlands 1944–45

The heavily urbanised western Netherlands endured a short but sharp famine during the final months of WW2, resulting in about 20,000 deaths^31,32^. The Dutch Hunger Winter remains the most important setting for famine-based research on foetal scarring. The births at risk were conceived in the six months between November 1944 and May 1945. Conceptions in the famine-affected area as defined by Stein et al.^10^ plummeted; the number of births 9 months later (i.e. between August 1945 and January 1946) totalled 12,804, compared to 23,891 between August 1944 and January 1945.

### Leningrad 1941–43

No firm estimate exists of famine-related excess mortality in Leningrad during the German blockade of 1941–44^33^. The number chosen here, 0.7 million, is almost certainly on the low side^34^. The famine was at its most intense in late 1941 and early 1942. The recorded number of live births in Leningrad plummeted from 79,411 in 1939 and 66,604 in 1940 to 12,987 in 1942^35,36^. The nadir was reached in October (62 births) and November (63 births). We put the number of births at risk in 1942–44 at 100,000, against an expected 240,000 in the absence of famine.

### Ukraine 1932–33

The number of deaths caused by the Great Ukrainian Famine of 1932–33 is also disputed. Conquest^37^ put excess mortality at 5.1 million, while former Ukrainian president Viktor Yushchenko^38^ described “a state-organised programme of mass starvation that in 1932–33 killed an estimated seven million to ten million Ukrainians, including up to a third of the nation’s children”. Several well-known accounts of the famine from a range of disciplinary perspectives are available^37,39–41^. The difference between the demographers Meslé et al.^42^ and Rudnytskyi et al.^43^—2.6 million and 3.9 million, respectively—derives largely from their estimates of emigration. In light of measures taken by the Soviet regime in early 1933 to force migrants back to their villages and to halt future migration, Meslé et al.^42^ assume that *voluntary* emigration in 1933 was “almost nil”, though, in reality, some people managed to escape from Ukraine^44^. Data on the stock of labour camp inmates from Ukraine at the beginning of 1934 suggests that *forced* out-migration in 1933, even allowing for mortality from hunger and disease in transit or in the camps, was probably closer to 0.1 million than 0.2 million^45,46^. In the absence of hard data, we propose an arbitrary compromise toll of 3.6 million excess deaths. Both estimates broadly agree on famine births, with Rudnytskyi et al.^43^ providing the important added detail that nine-tenths of excess deaths occurred between March and August 1933. This is corroborated by data on grain consumption per adult equivalent in the Kiev region: 230 grams in January–July 1933, 472 grams in August–September 1933^30^. The number at risk from scarring was probably less than the 0.6 million born in 1934; still, erring on the side of caution, we assume *B* = 0.6 million, or 0.25 million fewer than in a non-famine setting.

### Ireland 1845–50

The question addressed here has a particular resonance for Ireland, given the widespread but poorly substantiated perception that the Great Famine of the 1840s—or “the cumulative effects of British colonialization, the Great Famine, and various 20th-century development schemes”^47^ —led to a significant rise in the prevalence of mental illness and, in particular, schizophrenia, although the hard evidence for such an increase in lacking^7,48–52^.

The death toll exacted by the famine was about one million. The birth rate on the eve of the famine was about 39 per thousand, implying 280,000 births in 1845–46. Allowing for a decline in population from 8.6 to 6.6 million and a birth rate of 25 per thousand during the famine would entail about 0.9 million exposed births^53,54^.

## Method: calculating the cost

Our estimates of the extra cases of schizophrenia due to foetal exposure are described in Table 2. Two versions (Case_A and Case_B) are given. The first is based on calculating *B.d.p* for the five events described. The outcomes range from 65 extra cases for the Dutch Hunger Winter to about 0.2 million for the Chinese Great Leap famine. The numbers for the Netherlands are strikingly small, those for China ‘big’. But even the 0.2 million is modest compared to the contemporaneous death toll of 30 million in China.

**Table 2:** Excess famine mortality and cases of schizophrenia (in reverse chronological order).

Country	Period	Death toll (million)	Births at risk (1000s)	Case_A^a^	Case_B^b^	Ratio_A (%)	Ratio_B (%)
China	1959–61	30	40,000	200,000	160,000	0.67	0.53
Netherlands	1944–45	0.02	13	65	62	0.33	0.31
Leningrad	1941–43	0.7	100	500	400	0.07	0.06
Ukraine	1932–33	3.6	600	3000	2400	0.08	0.07
Ireland	1846–50	1	900	4500	3000	0.45	0.30

Ratio = Schizophrenia cases as percentage of excess deaths toll.

^a^Case_A estimates of the extra cases of schizophrenia using the increase in the odds of developing the disease (*d*), the prevalence of the disease in normal times (*p*), and the number of births at risk due to foetal exposure (*B*).

^b^Case_B follows Case_A’s estimates but assumes loss rates of 5% among schizophrenia cases for the Netherlands, 20% for China, Ukraine, Leningrad, and one-third for Ireland.

As noted, in using *B.d.p*, we do not make allowance for the impact of deaths in childhood and adolescence on the cohorts at risk. For example, how many of the 13,000 conceived during the Dutch Hunger Winter or the 900,000 conceived during the Great Irish Famine would have passed away before developing schizophrenia? Since schizophrenia occurs rarely before the age of 15, the answer hinges on the proportion of these cohorts dying before reaching that age. In the case of the Netherlands, a life table for 1938–1942 is available online^55^ at the *Human Life-Table Database* (*HLD*). suggests that the proportion was small—about 7%. Such tables are unavailable for the other countries discussed here but a sense of the cumulative mortality in Ireland in the 1840s may be obtained from the English life-table for 1841 (also available at the *HLD*), where two-fifths disappear by age 15. The 1841 period life table for England may not be a bad approximation for China in 1960 from a period perspective, i.e. during the famine itself, but the very rapid improvement in life expectancy thereafter suggests that we should be thinking in cohort, rather than period terms. And Banister and Hill’s life tables for China in 1964–1982 suggest that the proportion dying before the age of 15 in that period was only 12%^56^. We accordingly opt for a loss rate of 20% for China. Analogous data are lacking for Ukraine and Leningrad, but those for England and the Netherlands offer upper and lower bounds. Accordingly, in Table 2, Case_B assumes loss rates of 5% for the Netherlands, 20% for China, Ukraine, Leningrad, and one-third for Ireland. The resulting numbers in Table 2 should be seen as necessarily imprecise estimates that err on the side of caution.

How big was big? The final columns of Table 2 report the ratios of estimated schizophrenia cases to excess famine mortality. They are intended to give a better sense of the cost of exposure *in utero* relative to the broader demographic cost of our five famines. The higher ratios for the Great Leap Famine and the Great Irish Famine are explained by their longer duration. The low ratio for Leningrad is due to the drastic impact of the siege on fertility. Overall, however, contemporaneous costs in terms of the number of deaths dwarfed long-run costs in terms of the number of schizophrenia cases. This can be explained by the fact that prevalence in normal times was modest and that only people born during a limited period were exposed to the famine.

## Discussion

Added perspective is gained by comparing the number of extra schizophrenia cases to the total number of schizophrenia cases in each of these countries. Data on the prevalence of schizophrenia in China before 1990 are sparse, but the Global Burden of Disease study reckoned that in 1990, 3,551,298 people were suffering from schizophrenia in China, compared to 5,498,737 in 2019^57–59^. Assuming all cases of excess schizophrenia in the exposed cohorts manifest at age 15 (the 160,000 Case_B estimates in Table 2) were still alive in 1990, they would have accounted for <5% (i.e. 160,000/3,551,298) of all cases in 1990, and a dwindling proportion thereafter (<3% in 2019 on the assumption that nine-tenths of those born ca. 1960 were still alive then). In Ireland, the 3000 famine-induced cases would have accounted for one-tenth of the assumed 29,000 cases in 1861 (that is, assuming a prevalence rate of 0.5% in a population of 5.8 million). Presumably the ratio was generally lower in other famine settings covered by this study, due to their shorter durations. Calculating the outcomes for several different famines also highlights how the importance of foetal origins depends on the exact circumstances of a famine in terms of the birth rate, averted births, and duration.

In sum, a growing literature has highlighted the link between foetal exposure to malnutrition and adverse health in later life. Studies that use famines as natural experiments have advanced biomedical research substantially in this area. This research draws attention to a previously neglected welfare cost of famines. However, a misreading of its findings (as indicated by references both in the press and in academic outlets; see the introduction above) has prompted greatly exaggerated claims regarding the long-run impact of foetal exposure during famines. How big was the impact relative to the contemporaneous costs of famine? Using schizophrenia as a case study, this paper has shown that the data required to provide a rough answer to this question are available and that the impact was modest. Yet our results should not be construed as a claim that prenatal exposure is unimportant. Far from it; their point is simply that the resultant impacts are only small a part of the devastation caused by famine.

## Data Availability

All the data used are in the public domain.
